# Assembly of Bio-Nanoparticles for Double Controlled Drug Release

**DOI:** 10.1371/journal.pone.0074679

**Published:** 2013-09-06

**Authors:** Wei Huang, Jianfei Zhang, Harry C. Dorn, Chenming Zhang

**Affiliations:** 1 Department of Biological Systems Engineering, Virginia Tech, Blacksburg, Virginia, United States of America; 2 Department of Chemistry, Virginia Tech, Blacksburg, Virginia, United States of America; National Cancer Institute at Frederick, United States of America

## Abstract

A critical limiting factor of chemotherapy is the unacceptably high toxicity. The use of nanoparticle based drug carriers has significantly reduced the side effects and facilitated the delivery of drugs. Source of the remaining side effect includes (1) the broad final *in vivo* distribution of the administrated nanoparticles, and (2) strong basal drug release from nanoparticles before they could reach the tumor. Despite the advances in pH-triggered release, undesirable basal drug release has been a constant challenge under *in vivo* conditions. In this study, functionalized single walled carbon nanohorn supported immunoliposomes were assembled for paclitaxel delivery. The immunoliposomes were formulated with polyethylene glycol, thermal stable and pH sensitive phospholipids. Each nanohorn was found to be encapsulated within one immunoliposome. Results showed a highly pH dependent release of paclitaxel in the presence of serum at body temperature with minimal basal release under physiological conditions. Upon acidification, paclitaxel was released at a steady rate over 30 days with a cumulative release of 90% of the loaded drug. The drug release results proved our hypothesized double controlled release mechanism from the nanoparticles. Other results showed the nanoparticles have doubled loading capacity compared to that of traditional liposomes and higher affinity to breast cancer cells overexpressing Her2 receptors. Internalized nanoparticles were found in lysosomes.

## Introduction

Nanoparticle (NP) based drug carriers have been studied as a potential model for tumor diagnostic and treatment [1], [2]. The advantages include their ability of transporting high dose of toxic drugs or contrast agent specifically to the tumor, and gradually releasing the drug thereafter [3], and the ability of co-delivering a therapeutic cocktail (i.e. combinations of chemotherapy and immunotherapy [4], chemotherapy and anti-drug resistance agent [5]), which is critical for an effective cancer treatment. So far, the major efforts to improve nano-drug carriers fall into three categories, (1) using better particles (i.e. replace non-biocompatible particles with biodegradable ones) [6]; (2) shifting the *in vivo* distribution towards the tumor (i.e. use polyethylene glycol (PEG) coated, or targeting ligand grafted particles) [7]; and (3) eliminating basal drug release in the circulation (i.e. use advanced particles with pH [8], temperature triggered release [9]).

For intravenously administrated chemotherapies, the basal drug leakage from the drug carrier is a critical problem since the hindrances of in blood movement and lack of attraction to the target site could largely delay the arrival of NPs to the tumor. Studies have shown that, 24 to 72 hours post administration, the particles were still mostly trapped in the liver, spleen and kidney, few were found in the lung, skin and at the tumor site [7], [10], [11]. In addition, NP size, shape, rigidity, surface chemistry and the tendency of adsorption onto other surfaces are the known factors that can slow the *in vivo* transportation [10]. The accumulation of NPs at the tumor site is still mainly based on the enhanced permeability and retention (EPR) effect. Therefore, drug carriers with minimum basal drug leakage, preferably with accurately controlled release, are strongly needed.

A typical drug release from NPs is usually initiated with a burst phase (up to 50% of total loading) within the first a few hours post exposure, and followed with a slow release phase [4], [12], [13]. The burst phase corresponds to the release of drug molecules that are loosely associated with the particle, mostly located in the surface of the NPs. The slow release phase is resulted from the drug release from the inner core via diffusion and erosion (for biodegradable particles) [12]. When injected, the initial burst release occurs before most of the NPs can reach the tumor. This would result in an undesirable drug leakage into the circulation and the organs, causing toxic side effect to the body and loss of drug potency at the tumor. On the other hand, pH-sensitive NPs can theoretically eliminate those unwanted leakage [8]. After administration, the NPs encounter a series of decreasing pH gradient during their journey to tumor cells. Firstly, the NPs travel with the blood flow (pH 7.2) [14] and extravasate at the tumor site through EPR. The NPs that successfully get into the tumor (pH 6.5) [15] could then have close contact with the receptors located at the surface of tumor cells. The receptors recognize the grafted targeting ligand on the NPs and initiate a receptor-mediated endocytosis into tumor cells [4]. Internalized NPs are transported within endosomes to the lysosomes (pH decreases from 6.5 in endosomes to 4.6 in lysosome [15]). pH-sensitive NPs are engineered to only release the content into low pH environment so as to eliminate any premature drug leakage prior to the lysosome stage.

In this work, we present the elimination of in serum paclitaxel release by functionalized single walled carbon nanohorns (SWNHs) supported pH-sensitive immunoliposomes (NsiL). NsiL is a combination of two types of traditional NPs, namely SWNHs [16] and liposomes. Combined NPs have been described most recently and shown to inherit combined advantages of both NPs [17], [18], [19]. Here, functionalized SWNHs function as a hydrophobic drug carrier and the backbone of the NP. While immunoliposomes serve as a tunable boundary confining the drug before the NP enters the tumor cells and mimic the surface of bacteria that facilitates cell recognition and endocytosis. Properties of loading capacity, size distribution, morphology, in buffer and in serum drug release profile, cell binding and internalization rate of NsiL were studied in comparison with liposomes and SWNHs.

## Materials and Methods

### Materials

Lipid materials and mini extruder were purchased from Avanti Polar Lipid (Alabaster, AL). Monoclonal anti-ErbB2 mouse IgG2bk (clone 4B8) was purchased from Sigma-Aldrich (St. Louis, MO). Herceptin was a generous gift from Genentech (San Francisco, CA). Paclitaxel was purchased from LKT Laboratories (St. Paul, MN). Cell lines, culture related products were purchased from ATCC (Manassas, VA). Molecular probes were purchased from Invitrogen (Carlsbad, CA). Luna 5u C18 (2) reverse phase high performance liquid chromatography column was purchased from Phenomenex (Torrance, CA).

### Synthesis of Functionalized SWNH(-CH_2_-CH_2_-COOH)_x_

SWNHs were synthesized by Nd:YAG laser vaporization of graphite rods in an argon atmosphere at 1100°C as described elsewhere [20]. SWNHs were then functionalized with carboxyl groups by high-speed vibration milling. Briefly, a mixture of SWNHs and succinic acid acyl peroxide (1∶100 in mass) was vigorously shaken in a stainless steel capsule for 1.5 h. The ground ultrafine power was collected and washed three times with acetone and centrifuged to collect the sediment. Twenty minutes sonication was performed to dissolve the sediment in ultrapure water yielding solutions of SWNH(-CH_2_-CH_2_-COOH)_x_.

### Liposome Formula and Formation

PEGylated liposomes were made by extruding hydrated lipid mixture of 1,2-dioleoyltrimethylammoniumpropane (DOTAP), 1,2-dioleoyl-sn-glycero-3-phosphoethanolamine (DOPE), cholesterol (CHOL), and 1,2-dioleoyl-sn-glycero-3-phosphoethanolamine-N-[methoxy(polyethylene glycol)-2000] (ammonium salt) (DSPE-mPEG2000). For ‘DOTAP’ liposomes or 1,2-distearoyl-sn-glycero-3-phosphocoline (DSPC), DOPE, CHOL, and DSPE-mPEG2000 for ‘DSPC’ liposomes, the lipid mixture was extruded through polycarbonate membranes with pore sizes of 100 nm. Prior to extrusion, lipid cake was hydrated in Tris-HCl buffer (pH 7.4) that contains 0.9% NaCl, 5% dextrose and 10% sucrose, at 55°C with perturbation for at least 1 h. Extrusion was conducted at the same temperature and repeated 7 times. Detailed properties of the liposomes used are shown in Table 1. Wherein 1,2-distearoyl-sn-glycero-3-phosphethanolamine-N-[maleimide(polyethylene glycol)-2000] (ammonium salt) (DSPE-PEG2000-mal) will be added post formation of the liposomes in conjugation with the targeting ligand to ensure a certain orientation, which will be discussed in the ‘NsiL formation’ section. Rhodamin B (rhB) labeled NsiLs were made by adding DOPE-N-(lissamine rhodamine B sulfonyl) (ammonium salt) (up to 15%w/w) to the lipid mixture.

**Table 1:** Properties of lipid used in NsiL immunoliposome formulation.

Lipid	Head group net charge	Fatty acid tail	Molar ratio	Function
DOTAP/DSPC	+1/0	18∶1, 9 cis/18∶0	40%	Form cationic liposome; interact with negatively charged nanohorn [17]
DOPE	0	18∶1, 9 cis	33%	Form pH sensitive area in the lipid bilayer structure on NsiL [29]
CHOL	NA	NA	20%	Increase fluidity to lipid bilayer
DSPE-mPEG2000	0	18∶0	5%	Coat NsiL with proper amount of PEG; increase half life of NsiL in circulation
DSPE-PEG2000-mal	0	18∶0	2%	Covalently couple with anti-ErBb2 monoclonal mouse IgG

### NsiL Formation and Purification

Excessively high concentration of paclitaxel (1–5 mg/ml) in methanol was well mixed with SWNH(-CH_2_-CH_2_-COOH)_x_ powder with brief sonication. Methanol was then eliminated in a fume hood overnight. Paclitaxel loaded SWNH(-CH_2_-CH_2_-COOH)_x_ was washed in ultra-pure water 3 times and suspended in the hydration buffer. To determine the amount of drug loaded in the nanoparticles, an aliquot of drug loaded SWNH(-CH_2_-CH_2_-COOH)_x_ was dispersed in methanol with agitation. Samples were taken from the dispersion after 6 h of extraction for paclitaxel concentration measurement by high performance liquid chromatography (HPLC) equipped with Luna C18(2) reverse phase column (RPC) at UV 227 nm [21].

Paclitaxel loaded SWNH(-CH_2_-CH_2_-COOH)_x_ were encapsulated into liposomes as described previously [17]. Briefly, excessive liposomes were incubated with SWNH(-CH_2_-CH_2_-COOH)_x_. Three freeze and thaw cycles were applied using liquid nitrogen and a warm water bath. Nanohorn supported liposome (NsL) with high purity was collected at the bottom layer after 10 min centrifugation at 10,000 g in the presence of 10% sucrose.

Targeting ligand was conjugated to NsL particles through PEG. Briefly, anti-ErBb2 (Her2) monoclonal antibody (mAb) mouse IgG1 (Novus biologicals, Litteton, CO) was thiolated by Traut’s reagent in HBS buffer (25 mM HEPES, 140 mM NaCl, pH 7.4). Unreacted Traut’s reagent was removed by Sephadex G25 column on a fast flow liquid chromatography (FPLC) system. Immediately after concentration by an ultra-filtration centrifuge unit, corresponding (1∶1 molar) amount of DSPE-PEG-mal was added and the mixture was incubated overnight at room temperature. The product was added to the NsL suspension described in the previous section to a final ratio of 2% (Table 1) and incubated at 55°C overnight. The DSPE-PEG-mal mAb conjugates were added to pre-formed NsL particles to maximize the orientation with (1) the DSPE fatty acid tails inserted in the liposome bilayers and (2) the hydrophilic mAb end projecting away from the NsiL [22]. This orientation optimizes the exposure of mAb active binding sites resulting in improved efficiency of the receptor recognition, and a reduced chance of non-specific binding.

### Particle Characterization

Size distribution and zeta potential of NPs were analyzed on a Zetasizer Nano ZS (Malvern Instruments, Southborough, MA). Samples were freshly prepared before use by adding aliquots of NPs to 0.01 M sodium chloride buffer (5% dextrose and 10% sucrose in Tris-HCl buffer with a pH of 7.4) to make a solution with a lipid concentration of 0.01 mg/ml. During the test, samples were injected into a disposable capillary cell DTS1060 (Malvern Instruments, MA) and loaded onto the analyzer. Measurements were taken at 25°C with a material refraction index of 1.33 and viscosity of 0.8872 cp.

TEM images were taken for morphology study. Briefly, samples were deposited onto carbon coated copper grids for 5 min. 2% phosphotungstic acid was used for negative staining for 30 s. TEM images were taken by a JEOL JEM 1400 (JEOL Ltd, Tokyo, Japan).

### Drug Release Profile and Loading Capacity

Loaded drug (SWNH(-CH_2_-CH_2_-COOH)_x_ and NsiL) was extracted by dialysis and analyzed by high performance liquid chromatography (HPLC) [23]. Drug release profiles of NPs in aqueous solution and in serum at pH 7.2, 6.5 and 4.6 were measured by dialyzing equal amount of particles (0.1 mg SWHN or 0.4 mg lipid) against 20 ml corresponding buffer (citric acid at pH 4.6, sodium diphosphate at pH 6.5 and 7.2, each with 0.2% Tween 80) with agitation. Samples were collected at predetermined time point for a total of 30 days. After each collection, sample in the dialysis tubes was transferred to another 20 ml of fresh extraction buffer. Drug release studies in serum were conducted at 37°C.

For total loading capacity, 20 ml methanol was used for dialysis. Samples were thoroughly rinsed with ultrapure water and dialyzed at room temperature for 6 h. The extracted paclitaxel concentration was analyzed on an HPLC system equipped with Luna C18 (2) reverse phased chromatography column (at UV 227 nm).

### Cytotoxicity Assay

Cell toxicity of empty NPs was evaluated by cell proliferation assay. SK-BR-3 and BT-20 breast cancer cells were seeded onto 96 well plates at a concentration of 6 k/200 µl and incubated overnight (37°C, 5% CO_2_). Up to 0.64 µg lipid particles (or 0.1 mg SWNH) of aliquots NsL, SWNH(-CH_2_-CH_2_-COOH)_x_ and liposomes were added and incubated for 24 h. Cells were allowed to incubate with 1 mg/ml 3-(4,5-dimethylthiazol-2-yl)-2,5-diphenyltetrazolium bromide (MTT) in 0.1 M phosphate buffered saline (PBS) for 4 h. 200 µl dimethyl sulfoxide was used to dissolve the formazan crystals. Absorbance was measured on a Synergy HT Multi-Mode Microplate Reader (Bio Tek, Winooski, VT). Cell viability was calculated with an in-test calibration curve using the optical density of 560 nm subtracted by that of 670 nm.

### Cell Binding Assay

Affinity of NsiL particles to cells was measured by cell binding assay. Briefly, SK-BR-3 and BT-20 breast cancer cells were seeded onto 96 well plates at a concentration of 10 k/well and incubated overnight (37°C, 5% CO_2_). Up to 40 µg (lipid mass) of aliquots rhB labeled NsiL particles were added to incubate for 1 h. Each well was carefully rinsed and filled with a final volume of 200 µl 0.1 M PBS. Fluorescent intensities were measured immediately on the Synergy HT Multi-Mode Microplate Reader (excitation: 530, emission: 645, sensitivity: 40, optic position: bottom). NsL without attached targeting ligand and Herceptin NsiL were used for comparison.

### Cell Uptake Assay

The cell uptake efficiency was evaluated by confocal laser microscopy. Briefly, 3 ml SK-BR-3 cells were seeded onto glass bottom microwell dishes (35 mm petri dish, 14 mm microwell, 0.16–0.19 mm coverglass, MatTeck, Ashland, MA) at a concentration of 100 k/ml, incubated over night at 37°C, 5% CO_2_. 20 µl rhB labeled NsiL was added to the cell culture and incubated for 4–6 h. Culture medium was discarded and cells were rinsed 3 times with 0.1 M PBS. 30 µg calcein AM was added 15–30 min prior to the microscopy. Images were taken by a Zeiss LSM510 Meta (LSM TECH, PA).

## Results and Discussion

### Size Distribution and Zeta Potential of PEGylated DSPC Liposome, SWNH(-CH_2_-CH_2_-COOH)_x_ and NsiL

**Figure 1:**
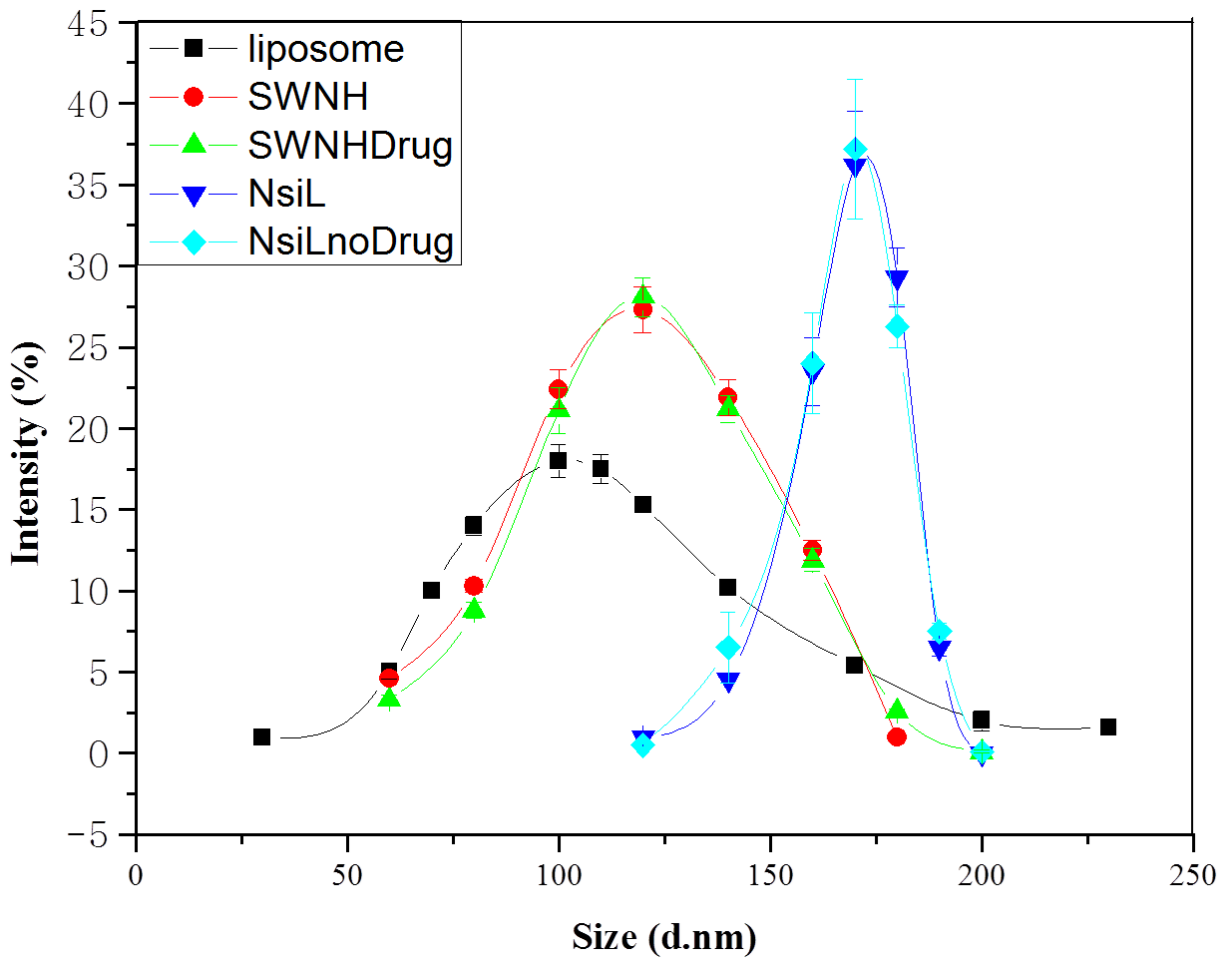
Size distribution of different nanoparticles. PEGylated DSPC lipsome (black), SWNH(-CH_2_-CH_2_-COOH)_x_ (red), paclitaxel loaded SWNH(-CH_2_-CH_2_-COOH)_x_ (green), DSPC NsiL (blue), and DSPC NsiL without paclitaxel (light blue). Sizes are shown in diameters (mean ± S.D., n = 45).

**Figure 2:**
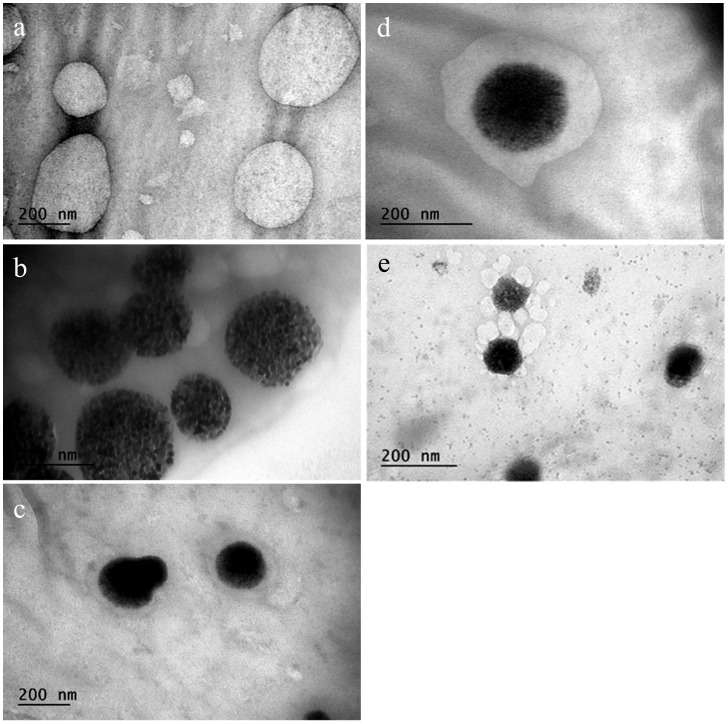
Morphology study of different nanoparticles by TEM. a, PEGylated DSPC liposomes; b, SWNH(-CH_2_-CH_2_-COOH)_x_; c and d, DSPC NsL; e, an intermediate step of a SWNH(-CH_2_-CH_2_-COOH)_x_ surrounded by multiple liposomes before the final one nanohorn one liposome structure. Immediately before TEM tests, samples were deposited onto carbon coated cupper grids and negatively stained with 2% phosphotungstic acid.

Size distributions of the NPs were analyzed by dynamic light scattering (DLS). Results are shown in Fig. 1. Mean diameters of 121 nm, 142 nm and 164 nm were observed for PEGylated DSPC liposome (Fig. 2a), SWNH(-CH_2_-CH_2_-COOH)_x_ (Fig. 2b), and NsiL (Fig. 2c, 2d), respectively. It is well recognized that sizes have significant influence on the *in vivo* distribution and loading capacity of NPs. Large NPs (d >200 nm) are known to be more vulnerable to the clearance by spleen and liver [24], not readily permeate to the tumorous capillaries [25], [26], nor, in some cases, good carriers of drugs due to the relatively smaller surface to volume ratio. On the other hand, small NPs (d <5 nm) leak out of the circulation at the kidney. It has been reported that administrated NPs with diameters around 150 nm tend to facilitate receptor-mediated endocytosis and had maximum accumulation in the tumor [27]. Noticing that the PEGylated liposomes and SWNH(-CH_2_-CH_2_-COOH)_x_ particles had relatively broad size distributions even though the mean diameter appeared within the desirable range. The purification process that has been applied to the NsiL NPs seemed to result in a narrow distribution for purified NsiL NPs as shown in Fig. 1.

Intuitively, the loading of hydrophobic drugs will lead to an increase of both the size and insolubility of the SWNH(-CH_2_-CH_2_-COOH)_x_ particles. However, the size distribution result of paclitaxel loaded nanohorns exhibited no significant difference from the unloaded ones (Fig. 1, green curve). In addition, no significant solubility decrease was observed during the particle assembly process, indicating the loaded drug has most likely entered the interior of nanohorns through the pores formed during the oxidation step. As a result, the NsiL particles with or without paclitaxel showed no observable deviation in size (light blue curve vs. blue curve).

Nevertheless, the mechanism of how SWNH(-CH_2_-CH_2_-COOH)_x_ gets encapsulated into liposomes is still unclear. One SWNH(-CH_2_-CH_2_-COOH)_x_ entering one liposome during the freeze and thawing process is unlikely the case, since the supported liposomes are much larger than the parental ones (Fig. 1). Li and Huang hypothesized that a supported double bilayer could form when two cationic liposomes approached one negatively charged core via membrane fusion [28]. Indications of such process were observed (Fig. 2e) in our tests as multiple DOTAP or DSPC liposomes (cationic liposomes) were observed to attach to the surface of a SWNH(-CH_2_-CH_2_-COOH)_x_ (anionic core). The attached liposomes were found merged into a larger liposome around the SWHNs most likely through membrane fusion during the freeze and thaw process.

The zeta potential values for DSPC PEGylated liposomes, SWNH(-CH_2_-CH_2_-COOH)_x_ and DSPC NsiLs were determined as 24.3, 34.6 and 22.5 respectively. To further investigate the colloid stability, we tested the size distribution of the same samples after 2 months of storage at room temperature (lipid concentration, 1.6 mg/ml). No aggregation was detected for all the samples (data not shown). This was most likely resulted from the use of PEGylated lipids in the assembly of the NPs.

### Morphology Study of NPs by TEM

Structures of nanohorn supported liposome (NsL, particles without targeting ligand) and NsiL were confirmed by transmission electronic microscopy (TEM). Spherical shaped PEGylated DSPC liposomes and SWNH(-CH_2_-CH_2_-COOH)_x_ were observed and shown in Fig. 2a and b, respectively. Structures of NsL NPs were shown in Fig. 2c and d. As shown, a SWNH(-CH_2_-CH_2_-COOH)_x_ was seen encapsulated in one liposome. The inter-space between SWNH(-CH_2_-CH_2_-COOH)_x_ and liposome could vary. DSPC NsiL showed identical morphology as NsL particles (not shown).

### Paclitaxel Loading Capacity

Total paclitaxel was extracted from PEGylated DSPC liposomes, SWNH(-CH_2_-CH_2_-COOH)_x_ and DSPC NsiL. Loading capacities were calculated from the corresponding HPLC peak area using a calibration curve. Drug loading efficiency (DLE, %w/w) was calculated as the ratio of mass of drug loaded to mass of NPs. Drug encapsulation efficiency (DEE, %w/w) was calculated as the ratio of mass of drug loaded to mass of drug added. DLE and DEE for DSPC NsiL were determined as 251.8% and 75.5%, respectively. In comparison, PEGylated DSPC liposomes showed a DLE of 118.4% and a DEE of 33.8%.

SWNHs have been reported as potential carriers for hydrophobic drug molecules. Besides the high loading capacity, loaded drug would have a long-term controlled release resulting from the slow diffusion rate of hydrophobic drug between graphene sheets [13]. Liposome, on the other hand, has seen more usages in delivering protein and gene based therapeutic agents and other water-soluble small molecules [2]. The loading of hydrophobic molecules in liposome is largely limited by their aqueous solubility. The use of SWNH(-CH_2_-CH_2_-COOH)_x_ particles has doubled the total loading of liposome particles. DLE and DEE of SWNH(-CH_2_-CH_2_-COOH)_x_ NPs were identical to DSPC NsiL indicating a rapid internalization of SWNH(-CH_2_-CH_2_-COOH)_x_ into liposomes, such that the drug loss during NsL assembly is negligible.

### In Serum Drug Release from NsiL

**Figure 3:**
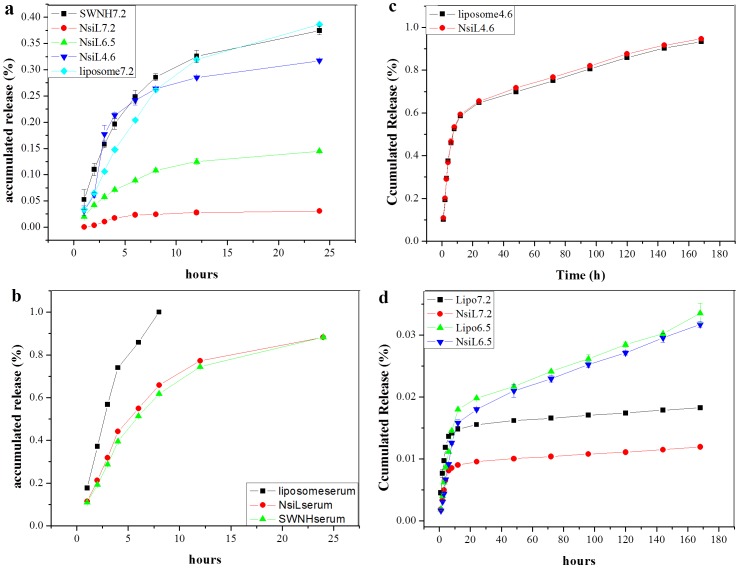
Paclitaxel release profile of different carriers. a, in buffer paclitaxel release from PEGylated DOTAP liposome, SWNH(-CH_2_-CH_2_-COOH)_x_ and DOTAP NsiL; b, in serum paclitaxel release from PEGylated DOTAP liposome, SWNH(-CH_2_-CH_2_-COOH)_x_ and DOTAP NsiL; c, in serum paclitaxel release from PEGylated DSPC liposome at pH 4.6; d, in serum paclitaxel release from PEGylated DSPC liposome at pH 7.2 and 6.5. All liposomes were PEGylated. All SWNHs were functionalized. Numbers in the legends indicate the pH.

**Figure 4:**
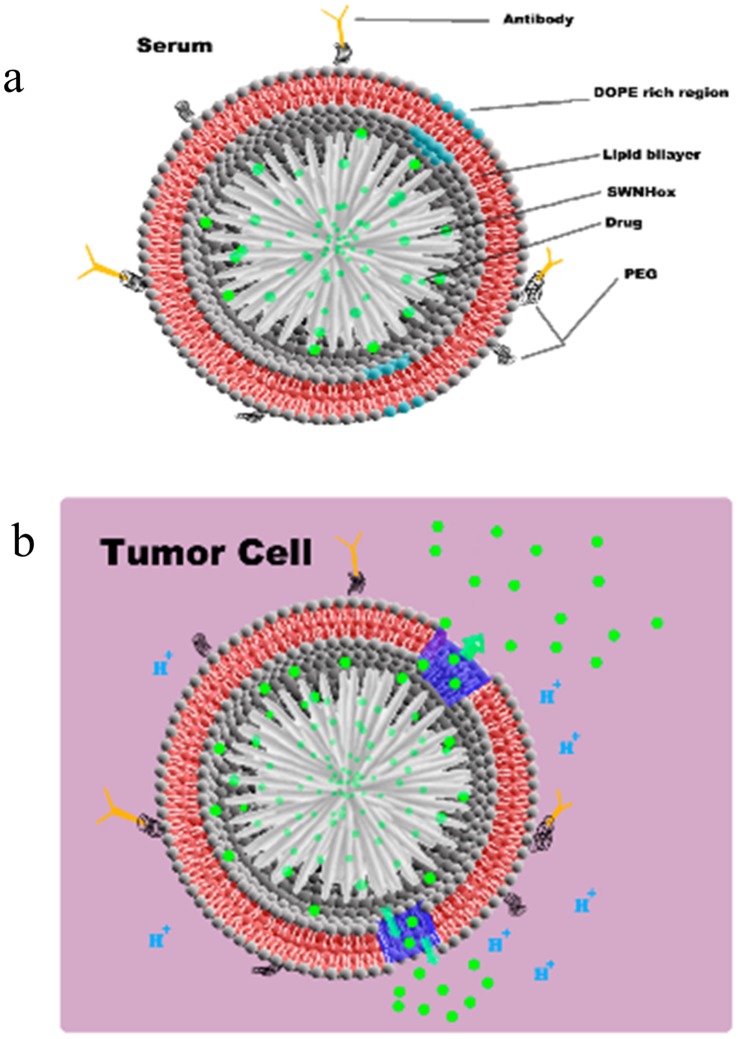
Structure and proposed drug release mechanism of NsiL. a, in serum; b, in tumor cells.

Drug releases from PEGylated DOTAP NsiL in saline buffer and in serum were tested. As shown in Fig. 3a, at pH 7.2 and room temperature in saline buffer, PEGylated DOTAP liposome (light blue) and SWNH(-CH_2_-CH_2_-COOH)_x_ (black) showed a 24 h cumulative release of 30%, in comparison to 2% of that from PEGylated DOTAP NsiL (red). A stronger release for NsiL NPs only started to show as the pH decreased to 6.5 and 4.6 (green and blue). The hypothesized mechanism is illustrated in Fig. 4. When the surrounding pH is neutral (i.e. 7.2 in the blood), the PEGylated liposome is intact and has a closed structure (Fig. 4a). The paclitaxel molecules (green dots) can slowly diffuse out of SWNH(-CH_2_-CH_2_-COOH)_x_ into the inter-space between SWNH and the liposome driven by concentration gradient. The paclitaxel accumulated in the inter-space could either diffuse back to the SWNH(-CH_2_-CH_2_-COOH)_x_ or continue diffuse to the outside of the NsiL. The latter is, however, largely limited by the presence of the lipid bilayer. Liposomes with varying resistance could be theoretically formulated by changing the lipid composition. We hypothesize that, using carefully formulated liposomes, the closed structure of the NsL NPs could be maintained for a given environment. The liposome used in this study consists 33% (mol/mol) DOPE, a pH sensitive phospholipid, which will have conformational changes and form defects on the liposomes under acidic pHs such as 4.6 (i.e. in lysosomes) [29]. Fig. 4b shows the release of drug under control of the diffusion out of SWNH(-CH_2_-CH_2_-COOH)_x_ and porous PEGylated liposome upon acidification mimicking the pH environment of that inside lysosomes of a tumor cell.

According to the hypothesized mechanism described above, the double controlled release (pH and diffusion) could eliminate basal drug leakage. To test this hypothesis, drug release from NsiL, SWNH(-CH_2_-CH_2_-COOH)_x_ and PEGylated DOTAP liposome were tested in serum at 37°C. Unexpectedly, NsiL and SWNH(-CH_2_-CH_2_-COOH)_x_ showed almost identical release profile (red and green respectively, Fig. 3b). This indicates, for the NsiL sample, the supported liposome failed to provide any containment to the paclitaxel molecules in serum at pH 7.2. On the other hand, leakage of the drug from PEGylated DOTAP liposome evidently is much more severe (black, Fig. 3b), indicating that, under physical condition, liposome alone provides almost no control on the release and the hydrophobic interaction between the drug molecules and SWNH plays an important role in controlling the release of the drug molecules. It is worth to note that the in serum release was conducted in the absent of macrophages or other RES components. To our best knowledge, opsonisation alone does not break the lipid bilayer [30]. We thus suspect it is the 37°C temperature that has induced the formation of defects in lipid bilayer and rendered the liposome permeable. DOTAP has a transition temperature (Tm) of 0°C. Under 37°C, DOTAP liposomes are in a melting state. Therefore, to minimize the undesired drug release, some temperature tolerable phospholipids (i.e. DSPC, Tm = 55°C) would be good substitutions of DOTAP.

PEGylated DSPC liposomes and DSPC NsiL were thus prepared. In serum release results showed a linear release of drug up to 90% in 7 days at pH 4.6 with a burst release of 60% within the first 10 hours for both NPs (Fig. 3c). Minimum releases from both NPs at pH 6.5 and 7.2 were observed and shown in Fig. 3d. Although the PEGylated DSPC liposomes showed almost identical drug release profile as that of DSPC NsiL under acidic pH, it is worth noting that at pH 7.2, NsiL NPs have half of the basal release of that from the liposome particles. In addition, NsiL NPs have the following advantages in drug delivery over liposome NPs: (1) NsiL has more than twice of the loading capacity as PEGylated liposomes; (2) PEGylated liposomes are nonetheless susceptible to electrostatic, hydrophobic and van der Waals forces [31]; (3) NsiL particles are self-stabilized through the electrostatic attractions between the negatively charged SWNH(-CH_2_-CH_2_-COOH)_x_ (at pH 7.2) and the positively charged DSPC head group (N+), which renders the NsiL more resistant to physical ruptures such as shear force and turbulence of blood flow and collisions with other components of blood (i.e. red blood cells, lipoproteins). Although it is out of the scope of this study, we suspect that under dynamic conditions as that in *in vivo* blood flow, NsiL NPs will perform much better than PEGylated DSPC liposomes in terms of basal drug release.

### Cytotoxicity Study

**Figure 5:**
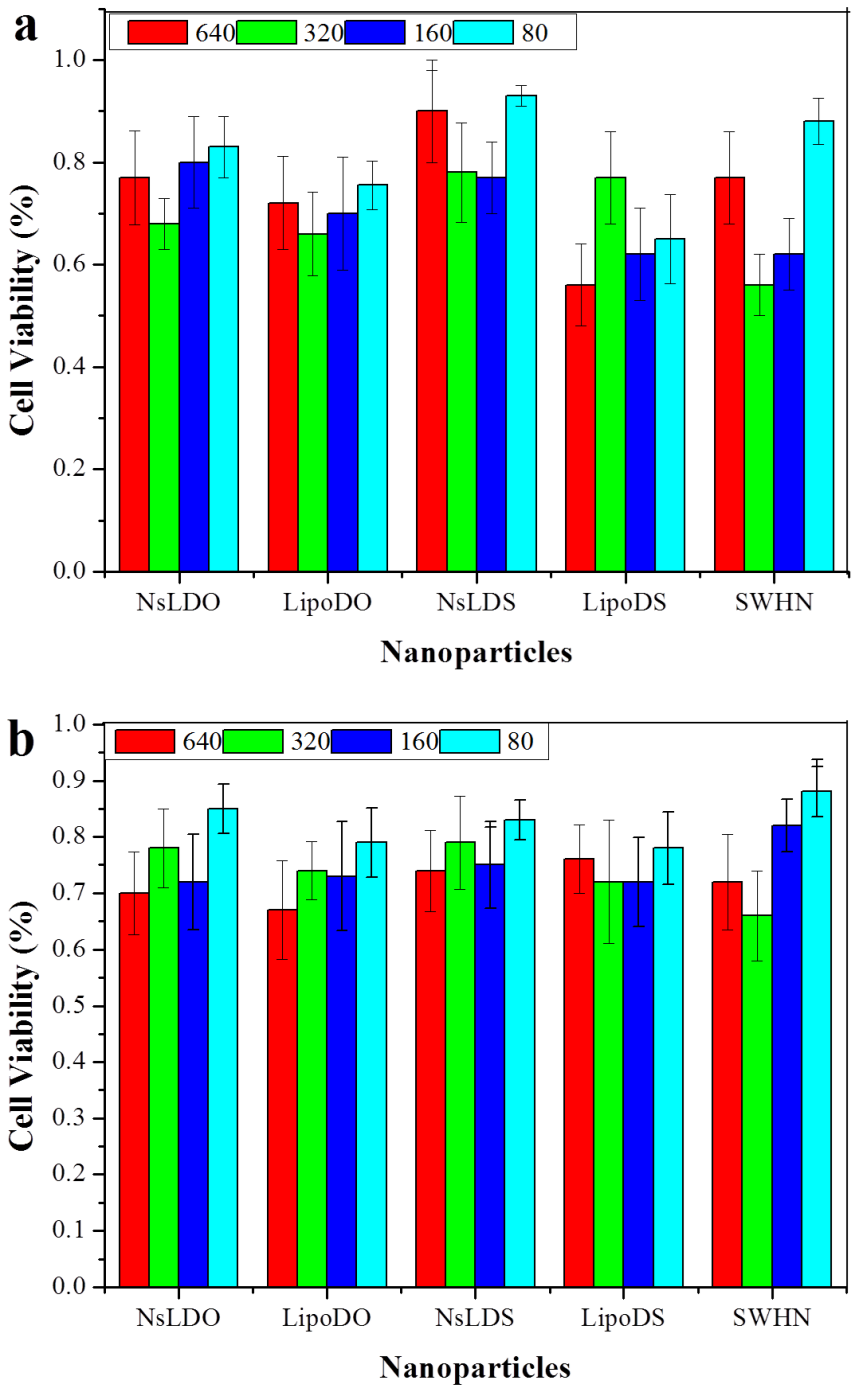
Study of NPs cytotoxicity by cell viability test. a, SK-BR-3 cells; b, BT-20 cells. Cells were treated with 640, 320, 160 and 80 µg/ml NPs (in lipid concentration). NsLDO and LipoDO are DOTAP NsL and PEGylated DOTAP liposomes, respectively. NsLDS and LipoDS are DSPC NsL and PEGylated DSPC liposomes, respectively. SWHN indicates SWNH(-CH_2_-CH_2_-COOH)_x_.

Cytotoxicity of NPs without paclitaxel and antibodies was evaluated by MTT assay. As shown in Fig. 5, both SK-BR-3 and BT-20 cells were incubated with up to 640 µg/ml NPs (in lipid concentration) for 24 h, and no significant cytotoxicity was observed for all tested NPs. Both PEGylated liposomes and SWNH(-CH_2_-CH_2_-COOH)_x_ have been previously reported as nontoxic [4], [32], [33]. As expected, the combined NPs appeared to be compatible with the breast cells. However, a noteworthy fact is that the *in vivo* fate of SWNH(-CH_2_-CH_2_-COOH)_x_ is still unclear. A possible degradation product, singlet or small cluster of nanohorn, will have a similar structure as a single-walled carbon nanotube, which has been reported to cause DNA damage, cell dividing interruption and inflammation [34]. Further study of *in vivo* long-term toxicity of SWNH(-CH_2_-CH_2_-COOH)_x_ is needed.

### Affinity of NPs to Her2-possitive and Negative Cells

**Figure 6:**
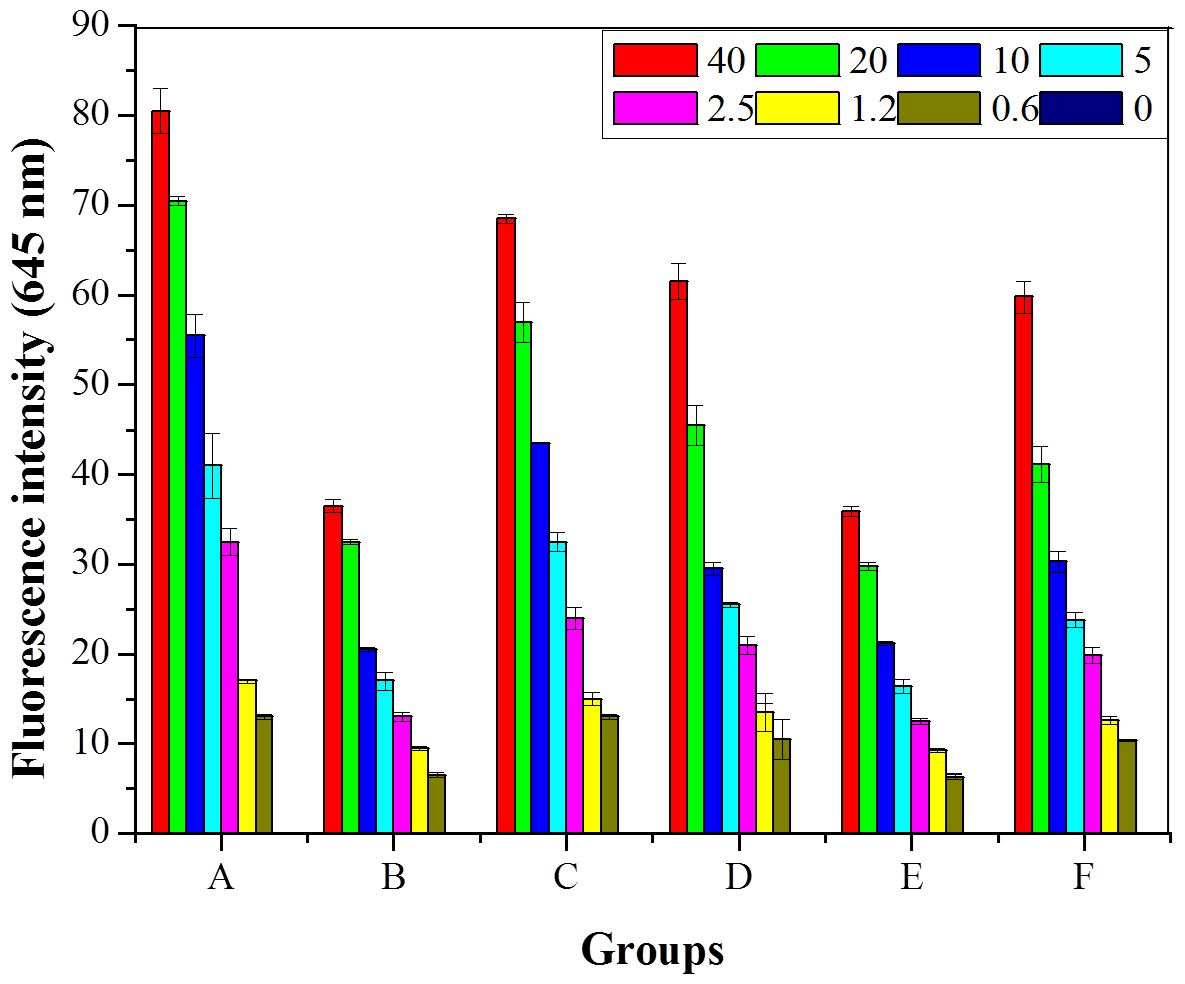
Cell binding affinity of different NPs. Different concentrations of particles were incubated with cell cultures, 40 µg/ml, 20 µg/ml, 10 µg/ml, 5 µg/ml, 2.5 µg/ml, 1.25 µg/ml, 0.625 µg/ml and blank control. A: NsiL with SK-BR-3; B: NsL with SK-BR-3; C: Herceptin NsiL with SK-BR-3; D: NsiL with BT-20; E: NsiL with BT-20; F: Herceptin NsiL with BT-20. NPs were labeled with rhB. Fluorescence emissions at 645 nm were measured.

Binding affinity of NPs to SK-BR-3 (a Her2-possitive cell line) and BT-20 (a cell line that has normal Her2 expression level) was evaluated. As shown in Fig. 6, NsiL attached with the anti-ErBb2 (Her2) monoclonal antibody (mAb) mouse IgG1 showed the strongest affinity to SK-BR-3 cells (group A), whereas the affinity of NsL particles without the targeting ligand (group B) and NsiL attached with Herceptin (a humanized monoclonal antibody of Her2 receptors [35], [36], [37]) (group C) to SK-BR-3 cells were relatively weaker. Evidently, NsL NPs also had certain degree of interaction with the cells, although the signal is only half as strong as NsiL at all tested concentrations. It is currently believed the targeting ligands provide no guidance for the NPs towards the tumor. They only facilitate the binding and internalization when NPs and cells get close to nanometer range [38], [39]. The difference between group A and group B seems to support this belief. Moreover, NsiL with anti-ErBb2 mouse mAb showed slightly higher affinity to the SK-BR-3 cells than the ones with Herceptin (Herceptin NsiL), and this can likely be attributed to the primary structural difference between the two antibodies.

Furthermore, the affinity of different NPs to BT-20 was also evaluated, as shown in Fig. 6 group D–F. Theoretically, since the cells do not overexpress Her2 receptors on their surface, all three NPs should show affinity to the cells similar to that between NsL and SK-BR-3 cells. It is not a surprise to see the almost identical signal intensities between group B and E for NsL NPs. Surprisingly, however, group D and F showed stronger signals than NsL particles (group E) at all concentrations, albeit the signals at corresponding concentrations are not as strong compared with that between these NPs and SK-BR-3 cells (group A and C). We hypothesize that the presence of proteins on NsiL and Herceptin-NsiL allows those NPs to engage in non-specific interactions with some proteins on the cells surface. Based on this hypothesis, attaching protein based targeting ligands on NPs really is a double edged sword, i.e. the ligands will enhance the interaction of the NPs with not only the targeted cells but also those cells the ligands are not intended to target [40].

Based on the over-expressed receptors, breast cancer cells have four main types, namely luminal, normal-like, Her2 and basal-like [41]. 20–30% of human breast cancer cells are Her2 (human epidermal growth factor receptor-2, Her2 or ErbB2) positive. Her2 is the most aggressive type of the four because of its high cell proliferation rate and propensity to early metastatization [42]. Dire prognosis and a less than 24–30 months median survival period are usually seen with Her2 positive patients [43], [44], [45], [46]. The NsiL NPs were designed to target Her2 positive breast cancer cells since the cells over-express Her2 receptor at the surface. Although the cell binding test results (Fig. 6) did not show a significantly preferential affinity of NsiL to Her2 positive cells, the targeting ligand tethered NPs still showed improved selectivity towards the targeted cells compared to NPs without any targeting ligands. It is worth noting that the cell binding test performed in this study is an *in vitro* test, in which different cell lines were cultured and evaluated separately. Thus, the test results were affinities without consideration of competition and a 3D cell network. Some works need to be done to better evaluate the NP distribution on different cells, such as testing on a matrix assisted *in vitro* tumor model composed of both Her2 positive and negative cells. On the other hand, the mAb and Herceptin used in this study were more than just targeting ligands. They are also a critical part of the potential treatment. Single-agent chemotherapy is usually not efficient for most of the cases [47]. Herceptin and other antibodies could attenuate the aberrant Her2 kinase-associated signal transduction, resulting in interrupted cell proliferation and metastatization [48], [49]. Co-delivery of paclitaxel (chemotherapy) and Herceptin (immunotherapy) has been shown effective by many [50].

### In Cell Distribution of NPs

**Figure 7:**
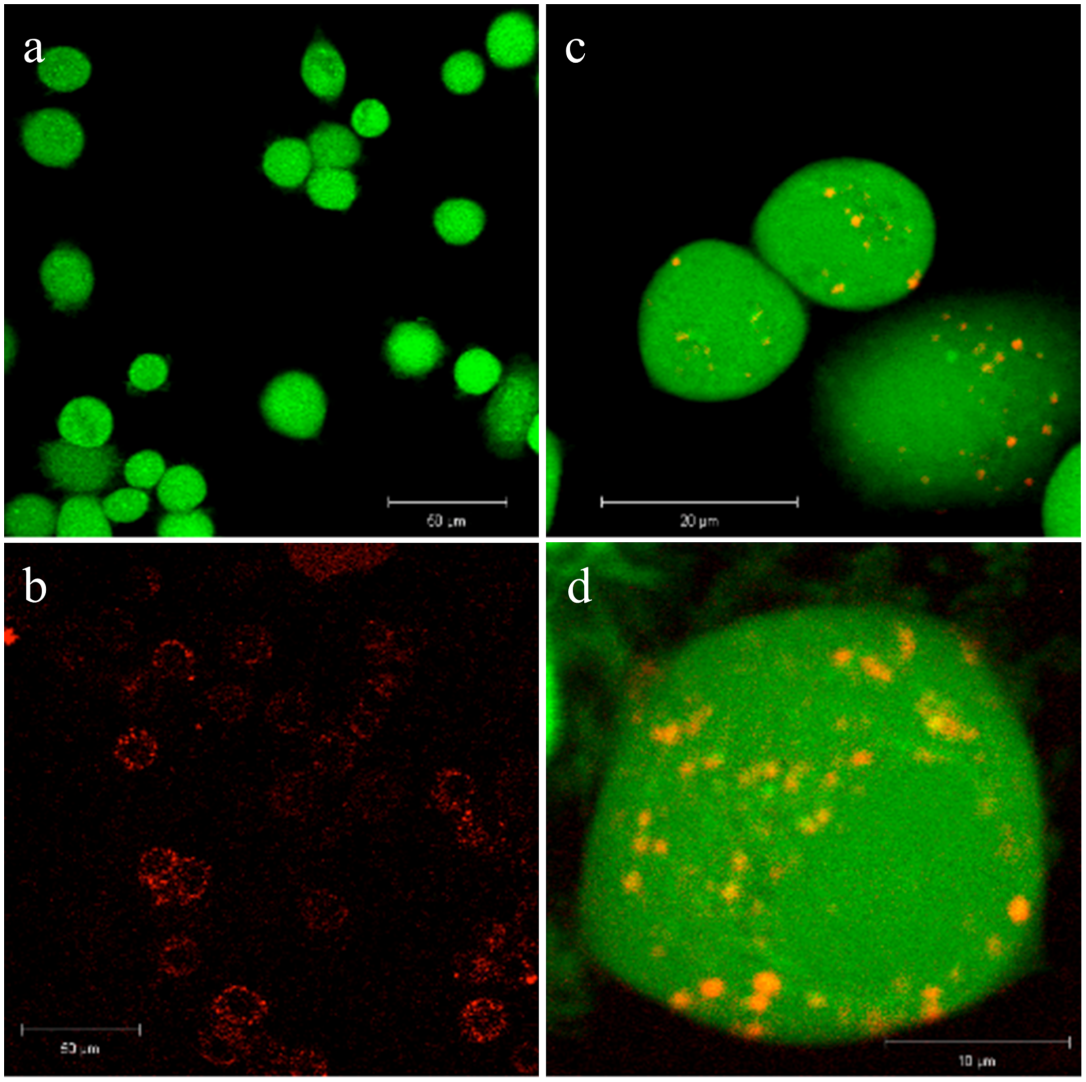
Cell internalization observed by confocal laser microscopy. a, stained SK-BR-3 cells with unstained NsiL; b, unstained SK-BR-3 cells with stained NsiL; c and d, co-stained NsiL in SK-BR-3 cells.

The internalized NsiL NPs were observed by confocal laser microscopy after a 6 h incubation of NsiL NPs with SK-BR-3 cells. As negative controls, either unstained NsiL or cells were used to prepare the sample and images were taken under stacks of FITC and rhodamine channel, as shown in Fig. 7a and b, respectively. Cellular distribution of NsiL NPs was shown in Fig. 7c and d. A lysosomal distribution was observed as particles scattered in the cytoplasm with slight higher density around the nucleus (Fig. 7d). This indicates that NsiL NPs were most likely internalized via receptor mediated endocytosis and delivered intracellularly into lysosomes for digestion.

### Other Considerations

It has to be pointed out that, *in vivo* drug availability always deviates from *in* *vivo* NPs distribution depending on the degree of undesirable premature leakage. The NsiL NPs could largely reduce the deviation, but will unlikely affect the NP distribution. Hence, to increase the proportion of NPs that reach the tumor has become a key for improved pharmacokinetics. Further studies are needed to improve the targeting specificity, stealth effect, and decrease the enhanced clearance upon repeatedly administration of PEGylated NPs (accelerated blood clearance phenomenon) [51].

The SWNH(-CH_2_-CH_2_-COOH)_x_ used in this study is highly soluble in water and was characterized elsewhere [52]. Surprisingly, the loading of paclitaxel (up to 75.5% DLE) showed no observable impact on the solubility of the SWNH(-CH_2_-CH_2_-COOH)_x_. In addition, identical size distributions were observed for nanohorn samples with or without paclitaxel (Fig. 1), indicating the minimal impact of drug loading on the properties of the nanoparticles.

NsiL could be used for many other applications. A magnetic resonance imaging (MRI) contrast agent Gd3+ containing (Gd3N@C80) trimetallic nitride template endohedral metallofullerenes (TNT-EMFs) has been loaded into SWNH(-CH_2_-CH_2_-COOH)_x_ with high MRI sensitivity and low toxicity [53]. PEGylated immunoliposomes could deliver the contrast agent containing SWNH(-CH_2_-CH_2_-COOH)_x_ to the targeting site allowing real-time monitoring of the *in vivo* distribution [54] or imaging or the pathogenic area within several days post infusion [53].

## Conclusion

A major advantage of NP based drug delivery is the precise targeting ability. However, over half of the loaded drugs were usually seen lost before the NPs can reach the tumor. The basal release in the circulation will cause reduced efficacy of the treatment and toxic side effect to the other parts of the body. In this study, NsiL NPs were assembled to eliminate the basal release of paclitaxel in the blood. SWNH(-CH_2_-CH_2_-COOH)_x_ are dahlia-like-shaped nanoparticle aggregates of single graphene tubules [16]. Functionalized SWHN has defects on the extensive surface and the surface is negatively charged at neutral pH. Paclitaxel was loaded to SWNH(-CH_2_-CH_2_-COOH)_x_, and cationic PEGylated pH sensitive, temperature insensitive liposomes was used to encapsulate the SWNH(-CH_2_-CH_2_-COOH)_x_ NPs. The NsL particle was self-stabilized through electrostatic interactions making it less susceptible to chemical and physical disruptions. Anti-Her2 mAb was grafted onto the NPs. Results showed the particles were formed with the described structure. The NsiL NPs had a narrow size distribution and high drug loading capacity. They could eliminate in blood release of paclitaxel based on a 2-level controlled release mechanism. Release of the drug was initiated upon acidification (around pH 4.3) and controlled by diffusion out of SWNH(-CH_2_-CH_2_-COOH)_x_ and the defects formed on liposomes. A composite release profile will contain 3 drug release stages, a minimum leakage in the blood, a burst release on entering lysosomes of tumor cells, and a slow linear release thereafter. *In vitro* tests showed NsL particles were low in cytotoxicity and active in cell binding and receptor mediated endocytosis.
